# Expression Analysis and Knockdown of Two Antennal Odorant-Binding Protein Genes in *Aedes aegypti*

**DOI:** 10.1673/031.010.14131

**Published:** 2010-10-05

**Authors:** Meryem S. Sengul, Zhijian Tu

**Affiliations:** ^1^ Department of Biochemistry, Virginia Polytechnic Institute and State University, Blacksburg, VA, 24061, USA; ^2^ Current address: Department of Biology, Bozok University, Yozgat, 66200, Turkey

**Keywords:** Gene knockdown, mosquito, olfaction, RNAi, Sindbis virus

## Abstract

The presence and expression of odorant-binding proteins (OBPs) in the olfactory organs suggest that they play an important role in mosquito olfaction. However, no direct evidence has been found for their involvement in the host-seeking behavior of mosquitoes. It is important to establish a method in which a loss-of-function test can be performed to determine the possible role of these genes in olfaction. In this study, a double subgenomic Sindbis virus expression system was used to reduce the expression of two *Obp* genes in *Aedes aegypti* L (Diptera: Culicidae), *AaegObp1* and *AaegObp2*. Quantitative real-time PCR analysis showed predominant expression of both genes in the female antennae, the primary olfactory tissue of mosquitoes. Moreover, at 11 days post virus-inoculation, the mRNA levels *of AaegObp1* and *AaegObp2* were significantly reduced in olfactory tissues of recombinant virus-inoculated female mosquitoes compared to that of controls by approximately 8 and 100-fold, respectively. These data suggest that the double subgenomic Sindbis virus expression system can be efficiently used to knockdown *Obp* gene expression in olfactory tissues of mosquitoes. We discuss the potential for a systematic analysis of the molecular players involved in mosquito olfaction using this newly developed technique. Such analysis will provide an important step to interfere with the host-seeking behavior of mosquitoes to prevent the transmission of diseases.

## Introduction

Mosquitoes are the most important vectors for human diseases, such as malaria, dengue fever, and yellow fever. Most female mosquitoes require a blood meal to complete their reproductive cycle and their bloodfeeding behavior enables the transmission of pathogens to their hosts. Olfaction plays a critical role in host-seeking behaviors of mosquitoes (Takken and Knols 1999). Current strategies to control the transmission of vector-borne diseases are ineffective. Thus, novel approaches are desperately needed to reduce the disease transmission including new ways to interfere with mosquito host-seeking behavior. In this respect, mosquito odorant-binding proteins (OBPs), which are small, water-soluble molecules that transport the hydrophobic odorants through the aqueous lymph of the sensilla to their receptors in the olfactory receptor neurons, represent good candidate targets for interference.

In Diptera, the physiological role of an OBP in *Drosophila melanogaster*, named LUSH, has been determined. It has been reported that LUSH mediates the perception of an aggregation pheromone, 11-*cis* vaccenyl acetate (VA), to elicit aggregation behavior of the fruit flies (Xu et al. 2005). Recently, it has been shown that LUSH was required for the VA sensitive receptor, Or67d, to respond to VA (Ha and Smith 2006). Unfortunately, no direct evidence has been found for specific functions of any mosquito OBPs. Functional assignment of mosquito olfactory genes will undoubtedly benefit from effective and efficient *in vivo* gene knockdown methods, which are currently lacking.

Transformation technology provides the ability to study heterologous gene expression and gene regulatory elements, and permits gene knockdown experiments designed to study gene function and perhaps to reduce the ability for mosquitoes to transmit diseases. Using transposable elements, such as *mariner* and *Hermes*, heterologous genes have been integrated into the *Aedes aegypti Aedes aegypti* L (Diptera: Culicidae) mosquito genome (i.e., Sarkar et al. 1997; Coates et al. 1998). However, the lack of high transformation frequency and effective screening methods causes researchers search for alternative tools for efficient molecular analysis. In this aspect, virus-derived infectious-clone technology provides a powerful alternative. The use of double subgenomic Sindbis (SIN) virus expression systems has shown successful manipulation of genes in mosquitoes. It has been used to introduce the gene of interest *in vivo* (Higgs et al. 1993; Olson et al. 1994; Kamrud et al. 1995; Rayms-Keller et al. 1995) and facilitate the expression of heterologous genes in mosquitoes (Hahn et al. 1992; Olson et al. 1994, 2000). Virus expression systems have also been used to silence gene expression in mosquitoes (Olson et al. 1996, 2002; Johnson et al. 1999; Adelman et al. 2001; Shiao et al. 2001; Tamang et al. 2004). However, it is not known whether olfactory genes of mosquitoes can be efficiently knocked down using this method.

SIN viruses are arboviruses (genus *Alphavirus*, family *Togaviridae*) that naturally cycles between mosquitoes of the *Culex* genus and the avian hosts (Taylor et al. 1955). They are enveloped viruses that replicate exclusively in the cytoplasm of the infected cells (Strauss et al. 1984; Strauss and Strauss 1994). SIN virus genome is a positive-sense, single stranded RNA and is about 11.7 kb in length. The 5′end is capped and the 3′ end contains a poly (A) tail. The genomic RNA of SIN virus is used as a template for the synthesis of a complementary (-) strand RNA, which in turn produces new genomic (+) strand RNA and a shorter subgenomic (+) strand RNA. Accordingly, the 5′ two-thirds of the genomic RNA is translated to produce the non-structural polyproteins for viral replication machinery and the 3′ one-third of the genome is translated to generate structural proteins, capsid (C) protein and envelope glycoproteins (E1 and E2). A noncoding region (NCR) exists at both 5′ and 3′ ends. It has been found that 3′ NCR contains repeated sequence elements (Strauss and Strauss 1994) that may play a role in host specificity by interacting with host proteins (Kuhn et al. 1991). SIN and other alphaviruses gain entry into vertebrate and invertebrate cells by receptor-mediated endocytosis (Strauss and Strauss 1994). The TE3′2J double subgenomic SIN virus was developed from infectious cDNA clones of the manipulated viral RNA genome of a mouse neurovirulent strain of AR339 SIN virus (Lustig et al. 1988; Hahn et al. 1992). The TE3′2J viruses contain a second subgenomic promoter between the structural protein coding region and the 3′NCR. The exogenous gene is inserted 3′ to this promoter that enables the expression (or silencing via RNA interference, RNAi) of the gene of interest.

Several *Obp* genes, including *Obp1* and *Obp7* genes in *Anopheles gambiae* (GenBank accession numbers: AY146721 and AY146742, respectively) and their possible orthologs in *Anopheles Stephensi* (GenBank accession numbers: FJ410801 and EU816361, respectively) show high levels of expression in the antennae and a general female-bias in their expression patterns (Xu et al. 2003; Biessmann et al. 2005; Li et al. 2005; Sengul and Tu 2008, 2010). The possible orthologues of *Agam*OBP1 and *Agam*OBP7 were identified in *Ae. aegypti*, which are *Aaeg*OBP1 and *Aaeg*OBP2, respectively (Ishida et al. 2004). More recently, *Aaeg*OBP1 and *Aaeg*OBP2 are also referred to as *Aaeg*OBP39 and *Aaeg*OBP27, respectively (Zhou et al. 2008).

Previous studies mentioned above suggest that *Anopheles Obp1* and *Obp7* genes are likely involved in female olfactory response and are good candidates for further investigation in different mosquito species. It is shown here the complete expression profile of the possible orthologue of these two genes in *Ae. aegypti* female mosquitoes. Moreover, the construction of double subgenomic SIN recombinant viruses that produce antisense transcripts of each of the two *Obp* genes is described. The double subgenomic SIN recombinant virus-inoculated mosquitoes showed dramatic decrease in the mRNA levels for both *Obp* genes at 11 days post virus-inoculation (p.i.). These genes are abundantly expressed in the female antennae compared to the other female mosquito chemosensory tissues, which may indicate a significant role for both *Obp* genes in mosquito olfactory behavior. These results provide the use of double subgenomic SIN virus expression system to effectively knockdown olfactory genes in the antennae of mosquitoes. The establishment of this efficient method to knockdown olfactory genes opens the door to future systematic analysis of the molecular players involved in mosquito olfaction and thus will facilitate the development of novel targets or compounds that interfere with mosquito behaviors that are relevant to disease transmission.

## Materials and Methods

### Cells and medium

Baby hamster kidney cells (BHK-21, ATCC no. CCL-10) and *Aedes albopictus* C6/36 cells (ATCC no. CRL-1660) were grown in Dulbecco's minimal essential medium (DMEM) containing 10% fetal bovine serum (FBS), L-glutamine, 100 U/ml penicillin, and 100 g/ml streptomycin and were maintained at 37° C and 28° C, respectively.

### Mosquitoes

*Aedes aegypti* (Liverpool strain) were reared at 28° C and 80% relative humidity, with a photoperiod of 12:12 L:D.

### Expression analysis of *Obp* genes by quantitative real-time PCR

Total RNA was isolated from antennae, maxillary palp and proboscis, legs, and body devoid of appendages from 5-day-old female mosquitoes. First-strand cDNA was synthesized from 0.8 µg of total RNA using oligo (dT) primers (Invitrogen, www.invitrogen.com). Real-time PCR was performed using the TaqMan probe-based chemistry with an ABI Prism 7300 Sequence Detection System (SDS; Applied Biosystems, www.appliedbiosystems.com). Briefly, 2 µl of cDNA was used with 9.25 µl nuclease-free water, 12.5 µl 2X TaqMan Universal PCR Master Mix (Applied Biosystems), and 1.25 µl 20X Assay mix designed by ABI in a 25 µl PCR reaction volume. Each assay mix included 5TAM (5′-carboxyfluorescein) and 3′non-fluorescent quencher (NFQ) labeled probe corresponding to a cDNA fragment that is separated by an intron, ensuring that only cDNA products were quantified. Real-time PCR for *AaegObp1* (EAT38681) and *AaegObp2* (EAT42273) genes and the internal control, 40S *ribosomal protein S7* gene (*AaegRps7*; EAT38624), were carried out using the following primer pairs and probes:

*AaegObp1* primerF:
  5′-GCACAAATGCTGGAAGGAGTCT-3′
*AaegObp1* primerR:
  5′-CCATGTTTCGGTTCTGCATTTCTT-3′
*AaegObp1* probe:
  5′FAM-ACCCCAAGCATTACTTC-3′NFQ
*AaegObp2* primerF:
  5′-CGGGCTCGTAGCAGATGTTAC-3′
*AaegObp2* primerR:
  5′-CGGGTAGCTCCAAATTGTCCTT-3′
*AaegObp2* probe:
  5′FAM-ATGGCCGCTCAAATC-3′NFQ
*AaegRps7* primerF:
  5′-CGCGCTCGTGAGATCGA-3′
*AaegRps7* primerR:
  5′-GCACCGGGACGTAGATCA-3′
*AaegRps7* probe:
  5′FAM-ACAGCAAGAAGGCTATCG-3′NFQ

The thermocycler program consisted of 50° C for 2 min, 95° C for 10 min, 40 cycles of 95° C for 15 sec and 60° C for 1 min. The goal was to determine the relative amounts of *Obp1* and *Obp2* mRNA. Thus, TaqMan assays were done in parallel for the control gene, *AaegRps7*, as well. All test samples and the controls were performed in triplicates, each from independent collections.

The ABI Assays-on-Demand setup was used for the TaqMan Gene Expression Assays. ABI does not recommend testing of amplification efficiency because their extensively tested assay design ensures near 100% (+/- 10%) efficiency and because commonly used testing methods tend to produce unreliable results (ABI Publication 127AP05-01). However, to be sure, we performed assay validation by checking the amplification curves (Bohbot and Vogt 2005). All TaqMan PCR data were analyzed using SDS Software based on the comparative method (ΔΔC_T_) (Livak and Schmittgen 2001). Briefly, for every sample, an amplification plot was generated showing the reporter dye fluorescence (ΔRn) at each PCR cycle. For each amplification, a threshold cycle (C_T_) was determined, representing the cycle number at which the fluorescence passes the threshold. This is how the C_T_ values of *AaegObp1*, *AaegObp2* and the control gene (*AaegRps7*) were determined. The C_T_ value of the control gene was then subtracted from the C_T_ value of the *Obp* gene giving the ΔC_T_ value. Then, the ΔC_T_ value of each sample was normalized to that of the calibrator sample (i.e., maxillary palp and proboscis was used as the calibrator for comparison of *Obp* gene expression in selected tissues of females; Tables 1 and 2) resulting in the determination of ΔΔC_T_ value. Finally, 2^-ΔΔCT^ values were calculated to estimate the fold variations in the mRNA levels of both genes in different tissues in females relative to the calibrator, maxillary palp and proboscis.

### Construction of *AaegObp1* and *AaegObp2* cDNA clones

**Figure 1A.**
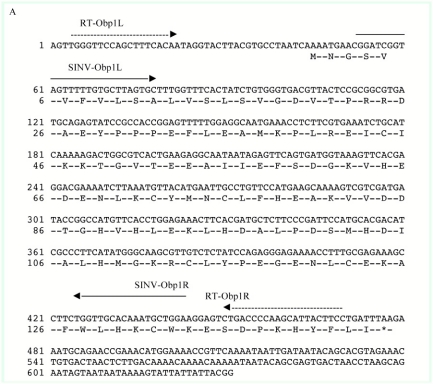


Total RNA was isolated from whole *Ae. aegypti* mosquitoes using TRIzol reagent (Invitrogen). DNase treated RNA was used to synthesize first-strand cDNA using oligo (dT) primers and the SuperScript II reverse transcriptase (Invitrogen, Carlsbad, CA, USA). The primer pairs used in PCR reactions included *Xba*I and *Pac*I restriction enzyme sites at the 5′ ends (Figures. 1A and 1B). Accordingly, the primer pair SJNV-Obp1R: 5′-TCTAGACAGACTCCTTCCAGCATTTG-3′ and STNV-Obp1L: 5′-TTAATTAA CGGATC GGTAGTTTTTGTGC-3′ were used to amplify a 401 bp of *AaegObp1* cDNA in the antisense orientation in a PCR with annealing temperature of 64°C for a total of 35 cycles. The primer pair STNV-0bp2L: 5′-TTAATTAAGGACAATTTGGAGCTAC CC-3′ and SINV-0bp2R: 5′-TCTAGATAACATCAGCC ATCAACAGG-3′ were used to amplify a 386 bp of *AaegObp2* cDNA in the antisense orientation in a PCR with annealing temperature of 61° C for a total of 35 cycles. The PCR products were cloned into pGEM-T-Easy vector (Promega, www.promega.com) and confirmed by sequencing (Virginia Bioinformatics Institute, https://www.vbi.vt.edu/).

### Construction of double subgenomic SIN recombinant virus plasmids

**Figure 2.**
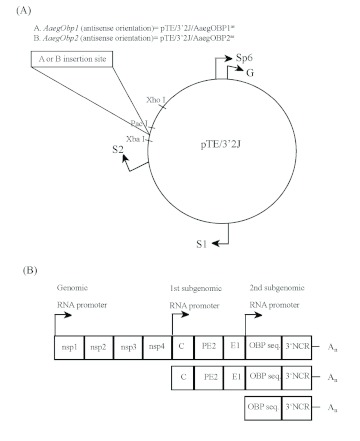
Recombinant pTE/3′2J viral plasmids and RNA transcripts produced upon viral infection. (A) Plasmid map of pTE/3′2J and construction of recombinant viral plasmids for *Obp* genes. The PCR amplified antisense transcript of *Aedes aegypti AaegObp1* and *AaegObp2* genes were cloned at *Xba*I and *Pac*I sites at the 3′ end of the second subgenomic promoter (S2), producing pTE/3′2J/AaegOBP1^as^ or pTE/3′2J/AaegOBP2^as^ recombinant viral plasmids. The location of the SP6 promoter used for *in vitro* transcription, genomic RNA promoter (G), 1^st^ subgenomic RNA promoter (S1), and 2^nd^ subgenomic RNA promoter (S2) are also indicated. (B) Representation of the genomic and subgenomic transcripts produced by the double subgenomic SIN recombinant viruses upon infection. Double stranded RNAs are produced during the virus life cycle. Parts of the construct are not to scale. Nsp 1–4: nonstructural protein 1–4, C: capsid, PE2: precursor envelope glycoprotein 2, E1: envelope glycoprotein 1, NCR: noncoding region. High quality figures are available online.

The pTE/3′2J viral construct was obtained from Dr. K.M. Myles (Virginia Polytechnic Institute and State University, Blacksburg, VA, USA) and has been previously described (Hahn et al. 1992). This plasmid was modified by adding a *Pac*I site downstream of the *Xba*I site (Figure 2) and the two sites were used for cloning purposes. Either *AaegObp1* cDNA or *AaegObp2* cDNA was cloned into the *Xba*I*/Pac*I digested pTE/3′2J plasmid in the antisense orientation. These recombinant plasmids are named as pTE/3′2J/Aaeg-OBP1^as^ and pTE/3′2J/AaegOBP2^as^, respectively. Antisense orientation of each cDNA insert in these recombinant plasmids was confirmed by PCR using primers complementary to the pTE/3′2J viral plasmid sequence flanking the inserted cDNA sequences.

### Double subgenomic SIN recombinant virus production

Double subgenomic SUNT plasmids were linearized at the *Xho*I site and their genomic RNA was transcribed *in vitro* using SP6 RNA polymerase (Promega) and capped with 7-methylguanosine (Ambion Inc., www.ambion.com). The RNA products were electroporated into BHK-21 cells with two consecutive pulses at 460 V, 725 ohms, and 75 F. Following incubation at 37° C for 48 h, the viruses were harvested and stored in aliquots at -80°C. The double subgenomic SFN recombinant viruses were propagated once in C6/36 cells and harvested from the medium 48 h and 72 h later. An aliquot of virus was titrated in BHK-21 cells using tissue culture infectious dose 50% end-points (TCJD_50_) assay.

### Mosquito inoculations with recombinant viruses

Five-day-old female *Aedes aegypti* mosquitoes were intrathoracically inoculated with double subgenomic SIN viruses pTE/3′2J (8.5 log_10_TCID_50_), pTE/3′2J/AaegOBP1^as^ (7.31og_10_TCID_50_) or pTE/3′2J/AaegOBP2^as^ (8.5 log_10_TCID_50_), respectively. Injected mosquitoes were held for 11 days at 28°C and 80% relative humidity and fed with sugar only. For all experiments, uninfected mosquitoes and pTE/3′2J viral plasmid inoculated mosquitoes were used as controls. Mosquitoes were collected at 3 days, 7 days and 11 days post virus inoculation (p.i.), homogenized and stored at -80° C until processing. Head tissues were also dissected from uninfected and double subgenomic SIN recombinant virus-inoculated mosquitoes at 11 days p.i.

### *Obp* gene expression in virus-inoculated mosquitoes

*AaegObp1* and *AaegObp2* mRNA levels in double subgenomic SIN recombinant virusinoculated mosquitoes were determined by using non-quantitative reverse transcription polymerase chain reaction (RT-PCR) and realtime PCR. The experiments included three sets of independent replicates for each uninfected, pTE/3′2J, pTE/3′2J/AaegOBP1^as^ and pTE/3′2J/AaegOBP2^as^ virus-inoculated mosquitoes. Whole mosquitoes were collected at 3 days, 7 days and 11 days p.i. Moreover, head tissues (antennae, maxillary palp and proboscis) of viral infected and control mosquitoes were collected in triplicates during three independent experiments at 11 days p.i. Total RNA isolation and cDNA synthesis were performed as mentioned above. cDNA synthesis reactions were also carried out in the absence of reverse transcriptase (-RT) in parallel for each sample as a control for the reactions. In addition, *AaegRps7* gene was used as an internal control. RT-PCR was performed using the following gene specific primer pairs:

RT-Obp1L:
  5′-TGGGTTCCAGCTTTCACAAT-3′
RT-Obp1R:
  5′-GGAAGTAATGCTTGGGGTCA-3′
RT-Obp2L:
  5′-TTATGCTGGCAGTTTTGCTG-3′
RT-Obp2R:
  5′-AGCTGGTAACATCAGCCATC-3′
RT-Rps7L:
  5′-GCTTTCGAGGGACAAATCG-3′
RT-Rps7R:
  5′-CAATGGTGGTCTGCTGGTTC-3′

Optimal annealing temperatures were 60° C for *AaegObp1*, 58° C for *AaegObp2*, and 61° C for *AaegRps7* genes and the amplification was performed for twenty-five cycles. PCR products were analyzed by 2% agarose-gel electrophoresis.

Furthermore, cDNAs from head tissues (antennae, maxillary palp and proboscis) of infected and control mosquitoes were used to compare their level of *Obp* gene expression by TaqMan based real-time PCR (see above for details). The primers used for real-time PCR ensure that the *Obp* RNA derived from recombinant SIN virus will not be amplified. The TaqMan assay primers and probes were described in the previous sections.

### Statistical analysis

ΔC_T_ values were used for analysis of real-time PCR data. One-way ANOVA with Tukey's post test was performed using GraphPad Prism version 3.00 for Windows (GraphPad Software, www.graphpad.com). Significance in comparisons was assumed if *P* < 0.05 is obtained in the appropriate test.

## Results

### *Aedes aegypti Obp1* and *Obp2* genes are likely orthologs of *Anopheles Obp1* and *Obp7* genes, respectively

A double subgenomic SUNT virus based *Obp* gene knockdown method was used for *Ae. aegypti* because the SUNT virus is known to be infectious to *Ae. aegypti* nervous systems. The *Ae. aegypti* orthologs of the *Anopheles Obp1* and *Obp7* genes (Xu et al. 2003; Sengul and Tu 2008, 2009) were used as targets for the *Obp* gene knockdown in *Ae. aegypti*. At the time this study was initiated, the genome annotation of *Ae. aegypti* (Nene et al. 2007) was not available, and few *Obp* genes were identified through conventional molecular techniques. Among those, sequence conservation indicated that *Aaeg*OBP1 (AY 189223; Ishida et al. 2004) showed the highest amino acid sequence identity (70%) to *Agam*OBP1, and *Aaeg*OBP2 (AY189224; Ishida et al. 2004) showed highest amino acid sequence identity (50%) to *Agam*OBP7. Phylogenetic analysis also suggested a possible orthology between *Aaeg*OBP1 and *Agam*OBP1, and between *Aaeg*OBP2 and *Agam*OBP7 (data not shown). *Aaeg*OBP1 and *Aaeg*OBP2 are also referred to as *Aaeg*OBP39 and *Aaeg*OBP27, respectively (Zhou *et al.*, 2008).

### Expression patterns in female *Ae. aegypti* mosquitoes

The ex pression of *Obp1 and Obp7* gene orthologs have been investigated in *Anopheles* (Biessmann et al. 2005; Li et al. 2005; Sengul and Tu 2008, 2009) and *Culex* (Pelletier and Leal 2009) mosquitoes. Our RT-PCR and subsequent cloning and sequencing results confirmed the splicing of introns that are encompassed by the RT-PCR primers (Figures 1A and 1B) in the two *Ae. aegypti Obp* genes, *AaegObp1* and *AaegObp2*. To determine the expression profile of these two genes, quantitative real-time PCR was performed using 5-day-old female *Ae. aegypti* mosquitoes, which are ready to take a blood meal. mRNA levels of the two genes were examined in the antennae, maxillary palp and proboscis, legs, and body devoid of appendages. An approximately 500-fold more abundant *AaegObp1* mRNA level was observed in the female antennae compared to that of the maxillary palp and proboscis (Table 1). The level of *AaegObp1* in maxillary palp and proboscis was significantly higher than female legs as well as bodies devoid of appendages, approximately 2.5-fold and 70-fold higher, respectively. *AaegObp2* mRNA level in the antennae was approximately 260-fold higher than that of the maxillary palp and proboscis (Table 2). The level of *AaegObp2* in maxillary palp and proboscis was significantly higher than female legs as well as bodies devoid of appendages, approximately 200-fold and 600-fold higher, respectively. These results clearly indicate that *AaegObp1* and *AaegObp2* are predominantly expressed in the antennae, the primary olfactory organs of mosquitoes, and may have a possible olfactory function. The expression levels of these two genes in the maxillary palp and proboscis are significantly higher than the levels seen in body devoid of appendages. Thus, these two genes may have biological importance in the maxillary palp and proboscis as well.

**Figure IB.**
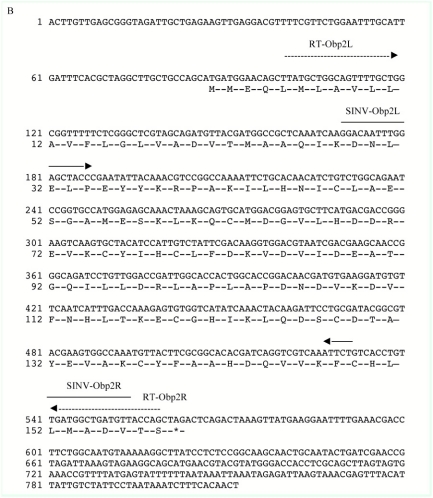


**Table 1.**
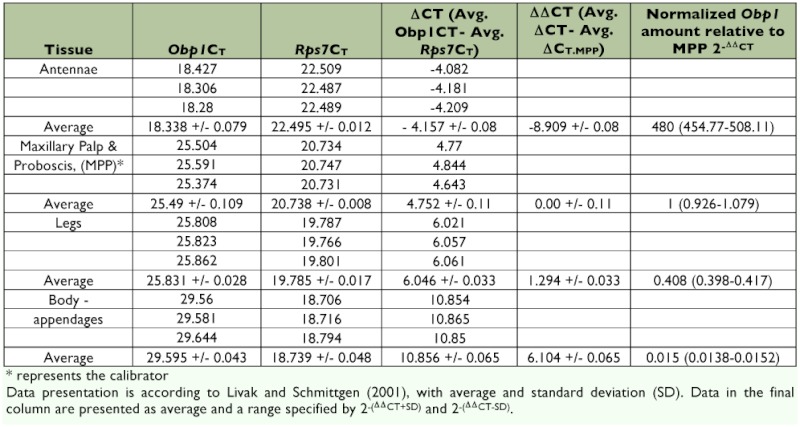
Relative expression of *AaegObp1* in female antenna, maxillary palp and proboscis, legs, and body devoid of appandages.

### Generation of the double subgenomic SIN recombinant viruses for *Obp* gene knockdown

A 401 bp *AaegObp1* cDNA and a 386 bp *AaegObp2* cDNA was cloned in antisense orientation using primers with *Pac*I and *Xba*I restriction enzyme sites (Figures 1A and 1B; see Materials and Methods). These cDNA sequences were inserted near the 3′ end of the second subgenomic promoter (S2) using the *Pac*I/*Xba*I sites in the pTE/3′2J plasmid (Figure 2A). The RNA was transcribed *in vitro* from the SP6 bacteriophage promoter of the pTE/3′2J recombinant plasmids and then electroporated into BHK-21 cells. Upon infection, genomic and subgenomic virus transcripts are produced as shown in Figure 2B. Double subgenomic SIN virus harvested after 48 h was further propagated by passage through *Ae. albopictus* C6/36 cells to obtain higher viral titers. Then, viruses were harvested after 48 h and 72 h and their titers were determined using tissue culture infectious dose 50% end-points (TCID_50_) assay. pTE/3′2J and pTE/3′2J/AaegOBP2^as^ viruses had similar growth patterns and attained a titer of 8.5 log_10_TCID_50_/ml at 72 h post-infection. The concentration of pTE/3′2J/AaegOBP1as virus stayed the same at 48 h and 72 h post-infection, reaching a maximum titer of 7.3log_10_TCID50/ml. As a result, double subgenomic SIN recombinant virus constructs were generated, one without an insert that was used as a negative control (pTE/3′2J) and those that were used as experimental samples producing antisense RNAs targeting either *AaegObp1* (pTE/3′2J/AaegOBP1^as^) or *AaegObp2* (pTE/3′2J/AaegOBP2^as^) mRNA.

### Double subgenomic SIN virus mediated RNA interference of *Obp* genes

**Figure 3.**
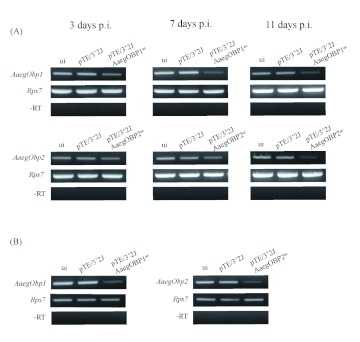
Non-quantitative RT-PCR analyses of *Aedes aegypti AaegObp1* and *AaegObp2* mRNA levels in uninfected, pTE/3′2J, pTE/3′2J/AaegOBP1^as^ or pTE/3′2J/AaegOBP2^as^ virus-inoculated *Ae. aegypti* female mosquitoes. (A) mRNA levels in whole mosquitoes at 3, 7, and 11 days after virus-inoculation (B) mRNA levels in the head tissues (antennae, maxillary palp and proboscis) dissected 11 days after virus-inoculation. High quality figures are available online.

Female mosquitoes were intrathoracically inoculated with either the pTE/3′2J, pTE/ 3′2J/AaegOBP1^as^ or pTE/3′2J/AaegOBP2^as^ recombinant SIN virus. On the 3rd, 7th and 11th days p.i., four groups of mosquitoes (uninfected, pTE/3′2J infected, pTE/3′2J/AaegOBP1^as^ infected, and pTE/3′2J/AaegOBP2^as^ infected) were collected and analyzed for *Obp1* and *Obp2* mRNA levels using whole mosquito preparations. Non-quantitative RT-PCR results showed progressive and apparent reduction of the *AaegObp1* and *AaegObp2* gene expressions in double subgenomic SIN recombinant virus-inoculated mosquitoes at 3, 7, and 11 days p.i. compared to that of the pTE/3′2J and uninfected controls (Figure 3A). In order to determine the mRNA levels of these *Obp* genes in olfactory tissues of *Ae. aegypti*, head tissues (including antennae, maxillary palp and proboscis) were collected from double subgenomic SUNT recombinant virus-inoculated mosquitoes at 11 days p.i. RT-PCR results showed a dramatic decrease for both gene transcripts in pTE/ 3′2J/AaegOBP1^as^ or pTE/3′2J/AaegOBP2^as^ virus-inoculated mosquitoes (Figure 3B). The internal control gene, *AaegRps7*, was robustly expressed for the tissue samples examined for all the RT-PCR reactions.

**Table 2.**
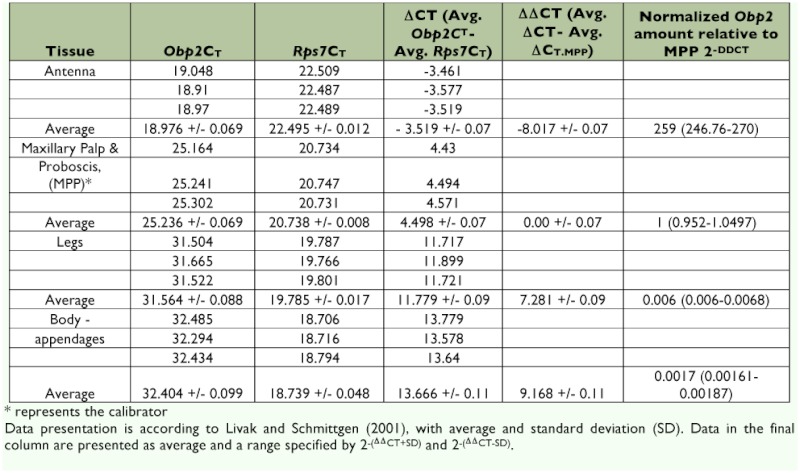
Relative expression of *AaegObp2* in female antenna, maxillary palp and proboscis, legs, and body devoid of appandages.

**Figure 4.**
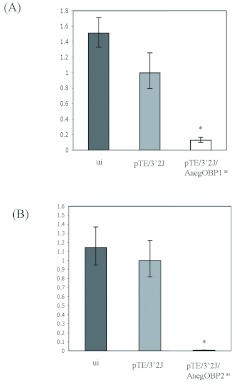
Real-time PCR detection of *Aedes aegypti AaegObp1* and *AaegObp2* mRNA levels in uninfected, pTE/3′2J, pTE/3′2J/AaegOBP1^as^ or pTE/3′2J/AaegOBP2^as^ virus-inoculated female *Ae. aegypti* mosquitoes at 11 days p.i. Total RNA from head tissues were used to synthesize cDNA for the detection of *AaegObp1* mRNA levels in pTE/3′2J/AaegOBP1^as^ virusinoculated female mosquitoes (A), and *AaegObp2* mRNA levels in pTE/3′2J/AaegOBP2^as^ virus-inoculated female mosquitoes (B), and compared to that of uninfected and pTE/3′2J control virus-inoculated females. Vertical bars represent the standard deviation representing the range of variation in three independent experiments. Asterisks indicate significant differences (*P* < 0.05). See Tables 3 and 4 for C_T_, ΔC_T_, ΔΔC_T_ and 2^-ΔΔCT^ values. High quality figures are available online.

In accordance with the RT-PCR data, realtime PCR analysis also confirmed that the transcript levels of *AaegObp1* and *AaegObp2* were decreased significantly in head tissues of double subgenomic SIN virus-inoculated mosquitoes (Figure 4). An 8-fold decrease in *AaegObp1* mRNA levels was observed in head tissues of pTE/3′2J/AaegOBP1^as^ virusinoculated mosquitoes (Figure 4A; Table 3); whereas, > 100-fold reduction was observed for *AaegObp2* expression in head tissues of pTE/3′2J/AaegOBP2^as^ virus-inoculated mosquitoes (Figure 4B; Table 4). As expected, there was no significant differences between uninfected and the control pTE/3′2J virus-infected samples. The observed reductions in transcript levels of both genes were indicative of knockdown in the olfactory tissues of *Ae. aegypti* female mosquitoes.

## Discussion

This study showed that *AaegObp1* and *AaegObp2* genes were expressed at higher levels in the antennae compared to the other chemosensory tissues of female mosquitoes of *Ae. aegypti*. This is consistent with what has been previously reported for the orthologues genes in *An. gambiae* (Biessmann et al. 2005; Li et al. 2005) and *An. Stephensi* (Sengul and Tu 2008, 2010). It is possible that these two *Obp* genes are likely involved in female olfaction in both *Aedes* and *Anopheles* mosquitoes. We also found that these genes are expressed at lower levels in the maxillary palp and proboscis of females. The low expression may be due in part to the fact that maxillary palp contains fewer sensilla than the antennae (McIver 1982). Although these two genes might be primarily involved in the olfactory behavior of female mosquitoes, they may also recognize different chemical molecules for a gustation-mediated behavior, such as sugar-feeding. Overlapping expression of *Obp* genes in maxillary palps and antennae have also been shown in *An. gambiae* (Biessmann et al. 2005), *An. stephensi* (Sengul and Tu 2008, 2010), *Cx. quinquefasciatus* (Pelletier and Leal 2009), and *D. melanogaster* (Galindo and Smith 2001). It has been found that 1-octen-3-ol, an attractant for *Ae. aegypti* mosquitoes, stimulates neurons on both palps (Lu et al. 2007) and antennae (Grant and O'Connell 1996). In *Drosophila*, the neurons of the palps and the antennae were stimulated by common chemical cues as well (de Bruyne et al. 1999). Thus, it is possible that maxillary palps and antennae have overlapping functions where an *Obp* gene may be involved in the perception of a common odorant in both tissues or it is involved in both olfactory and gustatory chemoperception.

Using *AaegObp1* and *AaegObp2* as targets, an effective method was established to knockdown *Obp* gene expression in *Ae. aegypti.* This method was based on dsRNA-mediated gene silencing using the double subgenomic SIN virus expression system. Real-time PCR results indicated that both gene expressions were reduced significantly in female head tissues (antennae and maxillary palp and proboscis) after infection with the double subgenomic SIN recombinant viruses when compared to those of the uninfected and control virus-inoculated samples. An 8-fold and >100-fold reduction were observed in transcript levels in head tissues of recombinant virus-inoculated female mosquitoes, which are the main sources of mRNAs for both genes (Tables 1 and 2). Given that the two *Obp* transcripts showed 259 and 480-fold higher levels in the antennae than in the maxillary palp and proboscis of females, the significant level of reduction in head tissues should mainly result from gene knockdown in the antennae. On the other hand, knockdown in maxillary palp and proboscis were likely masked by transcript levels in the antennae. In any case, this study showed a successful knockdown of two antennal *Obp* genes in the primary olfactory tissue, the antennae, of mosquitoes. To further assess the knockdown efficiency in the future, it is important to determine the protein levels of these OBPs in olfactory tissues.

**Table 3.**
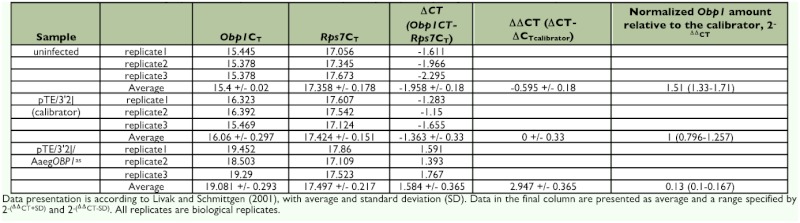
Relative expression of *AaegObp1* in olfactory tissues of uninfected, pTE/3′2J and pTE/3′2J/AaegOBP1^as^ infected mosquitoes.

**Table 4.**
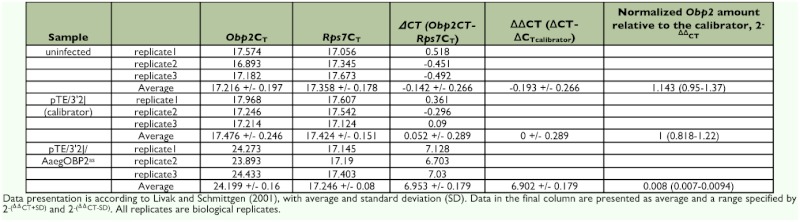
Relative expression of *AaegObp2* in olfactory tissues of uninfected, pTE/3′2] and pTE/3′2J/AaegOBP2^as^ infected mosquitoes.

As discussed above, the effectiveness of the gene knockdown in virus-inoculated mosquitoes is likely facilitated by RNAi-mediated inhibition of endogenous *Obp* gene expression. During the virus life cycle, dsRNAs would be produced which include the exogenously introduced sequences corresponding to the endogenous *Obp.* These dsRNAs, in turn, trigger the presumed RNA interference against the expression of endogenous mosquito *Obp* genes, and, thus, reduce the expression of these genes. As mentioned in the introduction, double subgenomic SIN virus based expression system has been successfully used to silence several immunity-related genes in *Aedes* mosquitoes. This study shows for the first time that the same effective strategy can be used to knockdown genes in olfactory tissues of mosquitoes. Thus, the power and the relative ease of using an infectious clone technology by simple manipulation can be harvested to allow the systematic investigation of the function of many olfactory genes by relatively efficient gene knockdown in mosquitoes.

Although insect OBPs are believed to be involved in odorant perception by transferring odorants to the olfactory receptor neurons, their specific functions have not been determined in mosquitoes. Our findings provide a potentially powerful new tool for assigning the functions of olfactory genes, such as *Obp* genes and olfactory receptor genes, in mosquitoes. Achieving this goal will likely be challenging and require extensive collaborations between mosquito geneticists and behavioral biologists. The reward, however, will be great improvement in understanding of the molecular basis of mosquito olfaction that will lead to possible new targets and compounds to interfere with mosquito behaviors that are relevant to disease transmission.

## Abbreviations

***AaegObp1***: *Aedes aegypti Obp1* gene;
***AaegObp2***: *Aedes aegypti Obp2* gene;
***AaegRps7***: *Aedes aegypti ribosomal protein S7 gene;*
**dsRNA**: double stranded RNA;
**OBPs**: odorant-binding proteins;
**p.i.**: post virusinoculation;
**RNAi**: RNA interference;
**SIN**: Sindbis
